# Tertiary lymphoid structures: new immunotherapy biomarker

**DOI:** 10.3389/fimmu.2024.1394505

**Published:** 2024-07-04

**Authors:** Fangyuan Yang, Jiahe Yang, Meijuan Wu, Cheng Chen, Xiaoyuan Chu

**Affiliations:** ^1^ Department of Medical Oncology, Jinling Hospital, Nanjing Medical University, Nanjing, China; ^2^ Department of Medical Oncology, Jinling Hospital, Affiliated Hospital of Medical School, Nanjing University, Nanjing, China

**Keywords:** tertiary lymphoid structures, cancer, immunotherapy, biomarker, cancer prognosis

## Abstract

Immunotherapy shows substantial advancement in cancer and is becoming widely used in clinical practice. A variety of biomarkers have been proposed to predict the efficacy of immunotherapy, but most of them have low predictive ability. Tertiary lymphoid structures (TLSs), the aggregation of multiple lymphocytes, have been found to exist in various tumor tissues. TLSs have been shown to correlate with patient prognosis and immunotherapy response. This review summarizes the characteristics of TLSs and the inducing factors of TLS formation, presents available evidence on the role of TLSs in predicting immunotherapy response in different cancers, and lastly emphasizes their predictive potential for neoadjuvant immunotherapy efficacy.

## 1 Introduction

Immunotherapy has revolutionized cancer treatment and rejuvenated the field of tumor immunology by enhancing patient’s immune response (1–3). Cancer immunotherapy provides unprecedented rates of durable clinical benefits for patients with different types of cancer, but the identification of potential responders before immunotherapy remains to be a challenge (4).

Tertiary lymphoid structures (TLSs), sometimes referred to as ectopic lymphoid structures, are organized aggregates of immune cells resembling secondary lymphoid organs (SLO, e.g. lymph nodes and spleen). Different from SLO, TLSs arise postnatally in nonlymphoid tissues under chronically inflamed environments, such as autoimmune diseases, allograft rejection, chronic inflammation and cancer (5). Although the mature TLSs have similar structure to SLO, the cellular makeup and molecular pathways involved in the process of TLS formation vary due to different local tissue context and disease. The presence of TLSs typically contributed to superior prognosis of cancer patients (6). Moreover, it has been reported that the presence of TLSs in tumor could predict an improved outcomes in cancer patients treated with immune checkpoint inhibitors (ICI) independently of PD-L1 status (7, 8). For example, the survival rate of TLS positive patients has been proved to be higher than that of TLS negative group in non-small-cell lung cancer (NSCLC) patients treated with ICI (9). However, not all TLSs positively contribute to the immune response against cancer, which may be attributed to distinction in TLS density, location or maturation status.

Therefore, this review aims to describe the characteristics of TLSs, including cellular composition, density, location, maturity and gene signature, and provide new insight into the predictive value for immunotherapy response in cancers.

## 2 Characterization of TLSs

**Figure 1:**
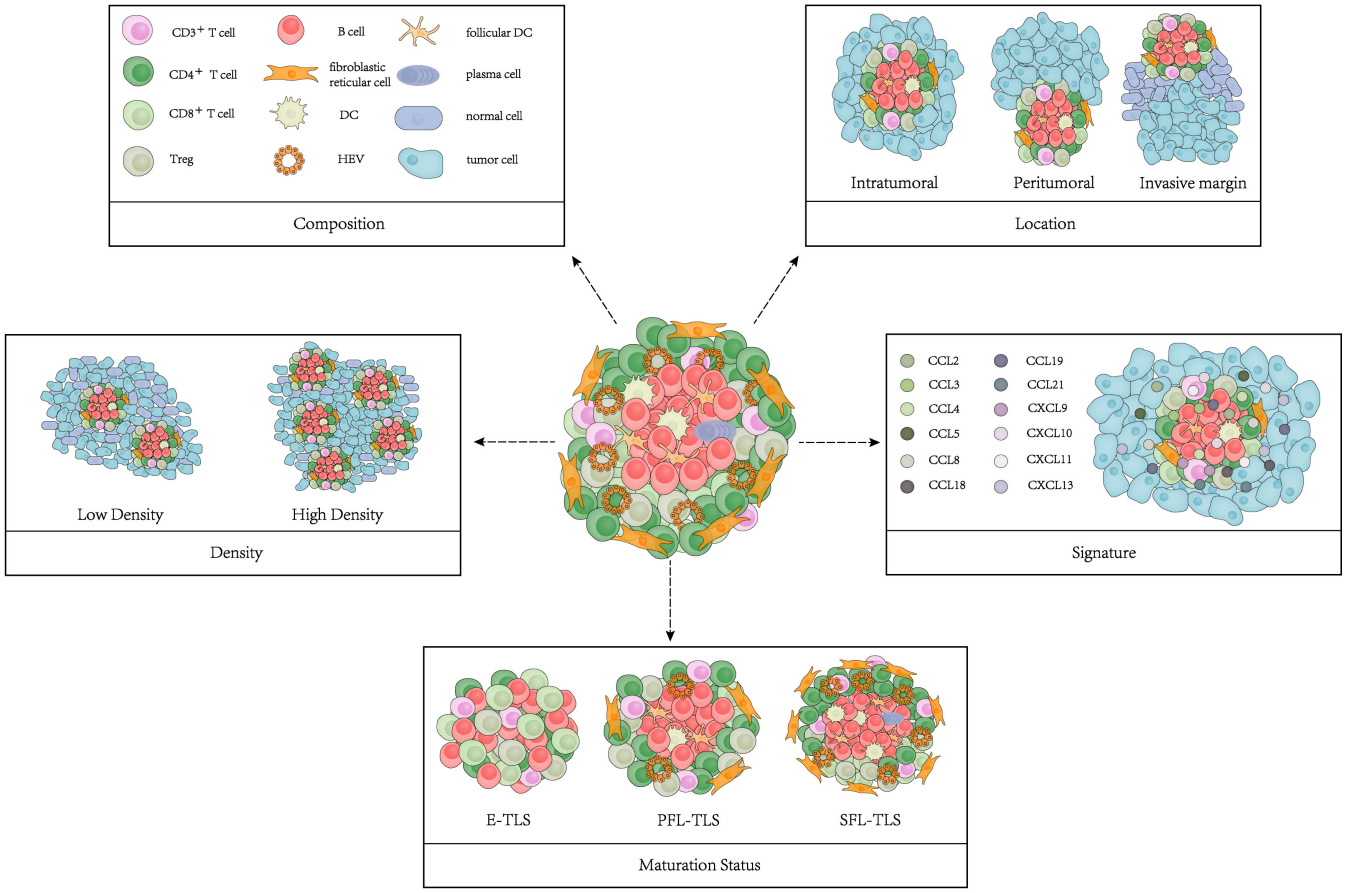
Model of TLS characteristics. TLS characteristics include composition, density, location, maturation status and signature.

The main characteristics of TLSs include composition, density, location, maturation status, and signature (**Figure 1**). We summarized the findings concerning the performance of these TLS features in cancer patient prognosis (10–16) (**Table 1**).

**Table 1:** Prognostic value of TLS characteristics in human cancers.

TLS features		Cancer types	Prognostic value	References
Composition	B cell	CRC	positive	(10)
	Mature DC	NSCLC	positive	(17)
	Plasmacytoid DC	Colon cancer	positive	(11)
	HEV	ICC	positive	(12)
		BC	positive	(13)
		CRC	positive	(14)
Density	High density	LSCC	positive	(18)
		HCC	positive	(19)
		OV	positive	(19)
		SKCM	positive	(19)
		UCEC	positive	(19)
Location	Intratumoral	HCC	positive	(20, 21)
		nmCRC	no prognostic value	(22)
		PDAC	positive	(23)
		OC	positive	(15)
	Peritumoral	HCC	negative	(24)
		nmCRC	positive	(22)
		PDAC	no prognostic value	(23)
		OC	positive	(15)
	Invasive margin	BC	positive	(25)
Maturity	Mature TLS	EC	positive	(16)
		PDAC	positive	(26, 27)
		ESCC	positive	(28)
		GC	positive	(29)
		nmCRC	positive	(30)
Signature	12-chemokines	ccRCC	positive	(31)
		LUAD	positive	(32)
		melanoma	positive	(33, 34)
		bladder cancer	positive	(34)
	CD79B, CD1D, CCR6, LAT, SKAP1, CETP, EIF1AY, RBP5, PTGDS	melanoma	positive	(35)

CRC, colorectal carcinoma; NSCLC, non-small-cell lung cancer; ICC, intrahepatic cholangiocarcinoma; BC, breast cancer; LSCC, lung squamous cell carcinoma; OV, ovarian cancer; SKCM, skin cutaneous melanoma; UCEC, uterine corpus endometrial carcinoma; HCC, hepatocellular carcinoma; nmCRC, non-metastatic colorectal carcinoma; PDAC, pancreatic ductal adenocarcinomas; OC, oral cancer; EC, endometrial cancer; ESCC, oesophageal squamous cell carcinoma; GC, gastric cancer; ccRCC, clear cell renal cell carcinoma; LUAD, Lung adenocarcinoma.

### 2.1 TLS composition

TLSs mainly consist of CD3^+^T-cell-rich region, CD20^+^B-cell-rich region, plasma cells (PCs), dendritic cells (DCs), and fibroblastic reticular cells (FRCs) (36, 37). Cytotoxic granule- expressing CD8^+^ T cells have been detected in surrounding T cell zone, as have CD4^+^ T cells orientated towards a Th1 cell phenotype and CD4^+^ regulatory T (Treg) cells (38). The inner B cell zone also includes CD21^+^ follicular dendritic cells (FDCs), germinal center (GC), CD83^+^ mature DCs, which are markers of mature TLSs (5). The vicinity of TLSs consists of lymphatic vessels, which express podoplanin and produce CC-chemokine ligand 21 (CCL21), and high endothelial venules (HEVs), which are characterized by the markers of peripheral node addressin (PNAd) (38). The follicles can further contain scattered CD68^+^ macrophages for clearance of apoptotic cells (5).

TLS formation could be induced by tissue-specific expression of chemokines. Heterogeneity of these driving factors may lead to differences in TLS components. Intriguingly, some specific cell types have been observed in the TLSs under certain conditions. LAMP^+^ DCs (mature DCs) have been considered to be reliable marker of TLSs in NSCLC as they were almost exclusively found in these structures (39). The density of LAMP^+^ DCs has been proved to be correlated with favorable clinical outcomes in NSCLC (17). A qualitative shift in the organization of TLSs has been found in Helicobacter hepaticum (Hhep) colonized mice in colorectal cancer (CRC), in which an increased presence of CD11c^+^ cells was found in the T cell zone of TLSs, consistent with an increase in DCs (40). A novel CD20^+^CD22^+^ADAM28^+^ B-cell subpopulation within TLSs has been reported to be present in ICI responders. These cells were named as ICI‐Responsive B cells (BIR) and were further identified as a subset of memory B cells that promoted the response to ICI therapy (41).

### 2.2 TLS density

TLS density varies among different individuals, even for a given cancer type and stage of the disease, emphasizing that individual tumor microenvironment (TME) can be more or less permissive to lymphoid neogenesis (42). High TLS density in tumors generally correlates with better prognosis. It has been reported that the expression of transcription factors related to adaptive immunity was significantly upregulated in TLS-high tumors (18). Patients with high TLS density exhibited significantly improved overall survival (OS) in hepatocellular carcinoma (HCC), ovarian cancer (OV), skin cutaneous melanoma (SKCM) and uterine corpus endometrial carcinoma (UCEC) (19, 43). In a lung squamous cell cancer (LSCC) cohort, high TLS density significantly correlated with improved progression-free survival (PFS) (16). Apart from primary tumors, a higher level of TLSs in metastatic tumors also showed significantly better OS (44–47).

### 2.3 TLS location

TLSs can be found in the peritumor, invasive margin and center of tumors (27). The location of TLSs may be important in predicting outcomes, associated with its function in the tumor immune response (48).

Some studies showed that patients with high level of peritumoral TLSs exhibited worse disease-free survival (DFS) and OS in breast cancer, cholangiocarcinoma (CCA), HCC and colorectal cancer liver metastases (CRLM) (24, 25, 49, 50). Zhang et al. found that the frequency of CD4^+^Bcl6^+^ T follicular helper (Tfh) cells was significantly increased in intratumoral TLSs compared to peritumoral TLSs in CRLM (50). However, one study found peritumoral TLSs as an independent and favorable prognostic factor in both OS and DFS for non-metastatic colorectal carcinoma (nmCRC) patients (22).

Unlike the dual role of peritumoral TLSs in prognosis evaluation, intratumoral TLSs have been proved to be associated with better outcome for cancer patients, including HCC and pancreatic cancer (20, 21, 23). Tumor tissues with intratumoral TLSs showed significantly higher infiltration of T and B cells and lower infiltration of immunosuppressive cells (23). TLSs at the invasive margin have also been validated as an important positive predictor of patient outcomes (48, 51).

### 2.4 TLS maturity

According to the cellular compositions, TLSs can be classified as follows: 1) Early TLSs (E-TLSs), distinguished by lymphocytic aggregates that lack a DC scaffold and vascularization; 2) Primary follicle-like TLSs (PFL-TLSs), also known as immature TLSs, comprised of T-cell and B-cell zones with FDCs but no GC; 3) Secondary follicle-like TLSs (SFL-TLSs), also known as mature TLSs, comprised of lymphatic vessels, isolated T-cell zones and a B-cell follicle with GC (30). Numerous studies have demonstrated that plasma cells, CD8^+^ T cells, and CD4^+^ T cells were more enriched in PFL-TLSs and SFL-TLSs (22, 28, 52).

Mature TLSs were reported to improve the prognosis of oesophageal squaenmous cell carcinoma (ESCC) and gastric cancer patients (28, 29). The cumulative risk of recurrence was significantly higher in patients with low SFL-TLSs in nmCRC (30). Mature TLSs supported antitumor adaptive immunity in pancreatic ductal adenocarcinomas (PDAC) (26, 27) and their formation was associated with a better prognosis in laryngeal squamous cell carcinoma (LSCC) with immunotherapy (52). Thus, mature TLSs have been confirmed to positively correlate with the prognosis and immunotherapy response of cancer patients.

### 2.5 TLS signature

Apart from H&E staining and immunohistochemistry (IHC) with multiplex selected markers to detect TLSs, transcriptomic analyses have also been developed to determine TLS-associated gene signatures in recent years (53). A 12-chemokine signature (including CCL2, CCL3, CCL4, CCL5, CCL8, CCL18, CCL19, CCL21, CXCL9, CXCL10, CXCL11, and CXCL13) is the most widely used signature for the quantification of TLSs in multiple solid tumors, including CRC, melanoma, HCC and breast cancer, etc (19, 33). Studies found that patients with high TLS signature displayed a better survival than those with low TLS signature, showing a marked association between TLS signature and the survival of cancer patients (32). Li et al. validated the clinical utility of the 12-chemokine TLS signature for predicting immunotherapy response by using two publicly available datasets. A high TLS signature score indicated strong immune infiltration and immune responses in both datasets (34). Another study by Xu et al. established TLS clusters in clear cell renal cell carcinoma (ccRCC) using machine learning algorithms and the 12-chemokine gene signature. Distinct differences were observed in survival, immune cell distribution, immunotherapy response among the TLS clusters (31). Cabrita et al. developed a gene signature (CD79B, CD1D, CCR6, LAT, SKAP1, CETP, EIF1AY, RBP5, and PTGDS) associated with TLSs in melanoma patients, which predicted clinical outcomes of melanoma patients treated with ICI (35).

## 3 Inducing factors of TLS formation

**Figure 2:**
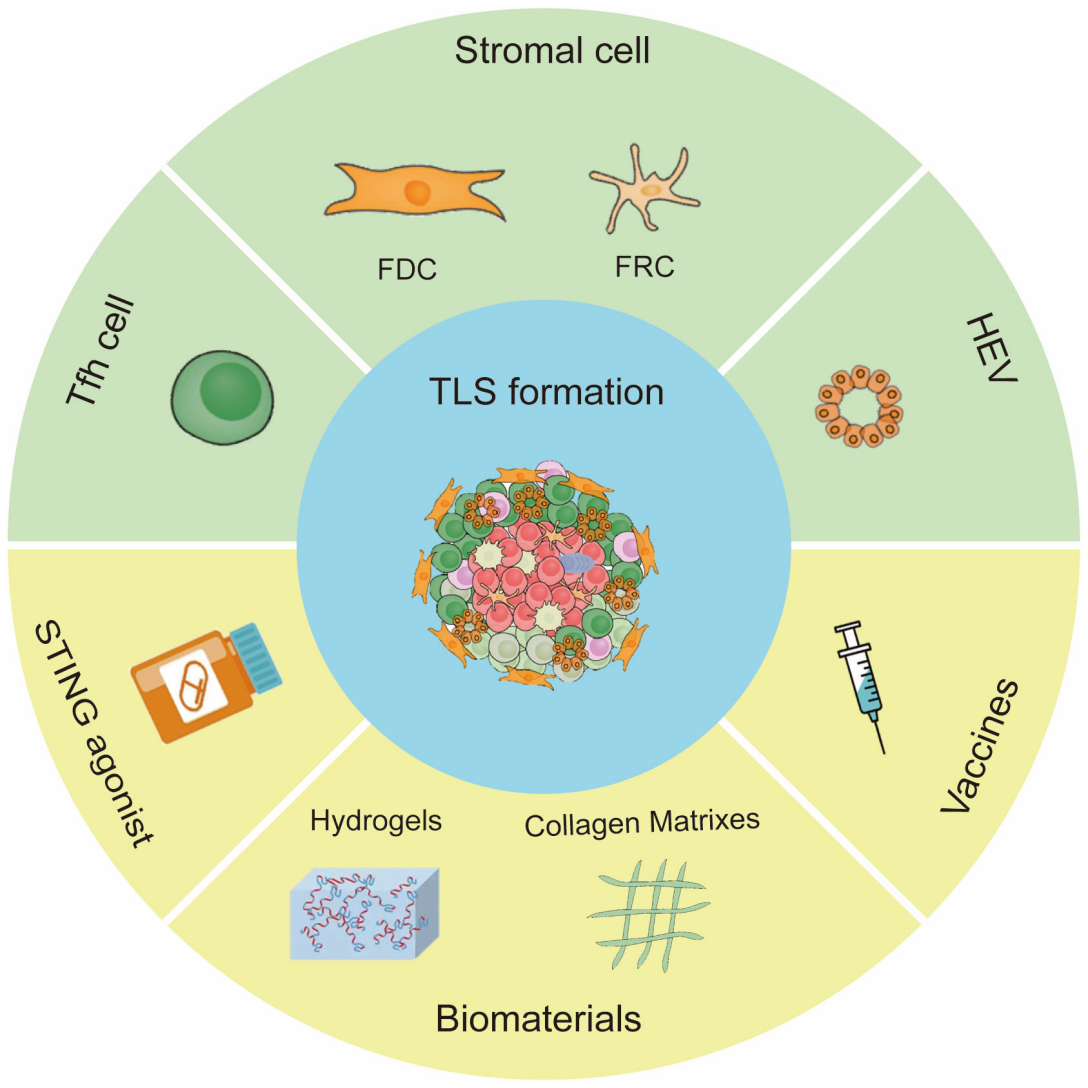
Inducing factors of TLS formation. The main cells for TLS formation are CXCL13 -expressing Tfh cells, stromal cells and HEVs. Methods to induce TLS formation include STING agonist, vaccine and biomaterials.

Tumor specific lymphocytes and stromal cells offer chemokines or cytokines required for TLS formation. Chemokines and lymphotoxins (LTs) are essential for the clustering of B/T cells and the development of lymphoid structures during TLS neogenesis. For example, CXCL13 and CXCL12 promoted the recruitment of B cells (54); CCL21 induced LTs expression on naive CD4 T cells and induced more organized infiltrates (55). In this section, we summarized the main cellular inducers of TLSs and potential pharmaceutical manners to induce TLS formation (**Figure 2**).

### 3.1 Cellular inducers of native TLSs

#### 3.1.1 CXCL13-producing T follicular helper cell

Tfh cells, a subset of CD4^+^ T helper cells, are specialized in helping B cell proliferation, survival, and differentiation, thus supporting antibody production and memory formation. The CXCL13-expressing Tfh cells are commonly co-localized with B cells in the tissue, allowing for Tfh cell to help the function of B cells and associated with the formation of TLSs (49, 56). Notably, enhanced generation of Tfh cells created a chemokine niche that promoted spontaneous assembly of TLSs at tumor beds (57). A recent study implied that CXCL13-producing CD4^+^ T cells were involved in the early stage of TLS formation (58), which consistent with another study indicating that TLS formation was dependent on CXCL13 signaling, potentially due to its effect on the recruitment of CXCR5^+^ B cells (59).

#### 3.1.2 Stromal cell

Stromal cells can differentiate into lymphoid tissue organizer (LTo) cells following stimulation by the lymphotoxin (LT) β receptor. LTo cells express chemokines and cell adhesion molecules that recruit and organize the B and T cell areas of the organ. LTo cells undergo further differentiation into the stromal cell subsets present in adult lymph nodes (LN) such as FRCs of the T zone, FDCs present in B cell follicles and GCs (60). Within the tumor microenvironment, the local cross-talk between immune cells and stromal elements leads to the production of a series of pro-inflammatory cytokines and TNF receptor family components that determine the formation of TLSs (51).

Zhu et al. created a mouse model of TLSs by implanting LN-derived stromal cells that express markers of FRCs. They found that TLSs were formed by expansion of stromal cells and gradual infiltration of B cells, CD4^+^ and CD8^+^ T cells (61). Nayar et al. demonstrated that fibroblasts could drive TLS formation in a melanoma model wherein they developed characteristics of LTo cells in response to TNF-receptor signal (62). Others found a CXCL13-CXCR5 chemotactic axis supported the proliferation of cancer associated fibroblasts (CAF) with LTo characteristics and TLS development (63, 64).

#### 3.1.3 HEV

HEVs express high levels of ligands for lymphocyte adhesion molecules, which facilitate the trafficking of T and B cells to the local tissue microenvironment. Tumor HEVs boost intratumoral lymphocytes infiltration, facilitating TLS maturation (65). Wang et al. induced endothelial differentiation from pluripotent stem cells and then constructed HEV-like organoids (HEVO). Upon transplantation into mice, HEVO promoted functional TLS formation by recruiting lymphocytes and enhanced antitumor activity (66).

### 3.2 Manners of TLS induction

#### 3.2.1 STING agonist

Multiple stimulators of interferon gene (STING) agonists have been developed for cancer therapy and have achieved promising results in pre-clinical work (67). Treatment with low-dose STING agonist ADU-S100 slowed tumor growth and promoted the formation of non-classical TLSs in murine B16 melanomas (68). Activation of STING within the TME increased the production of antiangiogenic factors and TLS-inducing chemokines and cytokines, resulting in improved vascular normalization (VN), enhanced tumor infiltration of CD11c^+^ DCs and CD8^+^ T cells and local TLS neogenesis (69, 70).

#### 3.2.2 Vaccines

Vaccines can stimulate body’s innate immunity and strong T cell response to combat infectious diseases and cancers. Lutz et al. first reported that granulocyte-macrophage colony-stimulating factor (GM-CSF)–secreting, allogeneic PDAC vaccine (GVAX) induced TLS formation and more T-cell infiltration in PDAC patients (71). Maldonado et al. observed memory T cells infiltration and TLS formation in vaccinated subjects with high-grade cervical intraepithelial neoplasias (72). TLS formation was also observed in resected stage IIB-IV melanoma after vaccination with AS15 and IFA (two cancer vaccines) (73).

Emerging of nanovaccines has improved targeted delivery, prolonged circulation and antigen presentation (74). Wen et al. demonstrated that the nanovaccine consisting of Epstein-Barr virus nuclear antigen 1 (EBNA1) and a bi-adjuvant of Mn^2+^ and cytosine-phosphate-guanine (CpG) formulated with tannic acid can foster TLS formation. The nanovaccine activated LT-α/β pathways, subsequently enhancing the expression of downstream chemokines CCL19/CCL21, CXCL10 and CXCL13 in the TME (75). Recently, researchers developed antigen-clustered nanovaccine (ACNVax) to activate immune cell. ACNVax plus anti-PD-1 antibody stimulated TLS formation and achieved long-term antitumor efficacy (76).

#### 3.2.3 Biomaterials

Recent studies revealed that some biomaterials not only serve as drug carriers, but also have intrinsic immunoregulatory effects (77). Collagens are major components of the extracellular matrix of connective tissues (78). Suematsu et al. initially used a collagen sponge biomatrix embedded with LTα-expressing stromal cells and transplanted *in vivo* to induce structures similar to TLSs (79). Subsequently, TLSs were also successfully induced using collagen sponge scaffolds contain chemokines and soluble RANK ligand (80).

Hydrogels are three-dimensional crosslinked polymer meshwork. With its properties, hydrogels can extend residence time as drug vector and are suitable for biomedical applications (81). Hydrogel preparations with BAFF-producing stromal cells and IL-4 promoted GC-like reaction in B cell and antibody class switching *in vitro* (82, 83). Another study described that STING-activating hydrogel (ZCCG) facilitated the formation of TLSs by recruiting immune cells and enhanced antitumor immunity (84).

## 4 The role of TLSs in predicting immunotherapy response

ICI plays an important role in cancer immunotherapy, and sustains a long-term immune response for cancer patients. The two leading ICI approaches are anti-PD1/PD-L1 and anti-CTLA-4 antibodies (85). ICI treatment induced the expansion of CD8^+^ T cells which was not observed before treatment, indicating that ICI response was driven by incoming T cells. TLSs may be instrumental to restart antitumor defense during ICI treatment by mounting a fresh adaptive immune response using incoming B and T cells (36). Studies have demonstrated that TLSs could serve as potential biomarker in predicting response to ICI therapy (86) (**Table 2**).

**Table 2:** Response rates to immunotherapy based on TLS status.

Cancer type	Treatment	Method	Percent of patients with positive TLS	Response rate in patients with positive TLS	References
GI	anti PD-1	IHC, TLS signature (12-chemokine signature)	NR	NR	(29)
Melanoma	anti CTLA-4	IHC, TLS signature (CD79B, CD1D, CCR6, LAT, SKAP1, CETP, EIF1AY, RBP5, and PTGDS)	NR	NR	(35)
NSCLC	anti PD-1	H&E	11/20 (Intratumoral)	11/11	(86)
	anti PD-1 plus chemotherapy	H&E, IHC	34/40 (Intratumoral)	17/34	(87)
CCA	anti PD-1 plus chemotherapy	H&E, IHC, TLS signature (PAX5, TCL1A, TNFRSF13C, and CD79A)	NR	NR	(88)
ESCC	anti PD-1	H&E, IHC, TLS signature (POU2AF1, LAMP3, CD79A, and MS4A1)	29/34 (Peritumoral)	9/29	(89)
HNSCC	anti PD-1	TLS signature (LAMP3, CCL2, CCL3, CCL4, CCL5, CCL18, CCL19, CCL21, CXCL9, CXCL10, CXCL11, CXCL13, and CXCR4)	NR	NR	(90)
STS	anti PD-1	H&E, IHC, TLS signature (CXCL12 and CCL18)	48/24 (Intratumoral)	14/30	(91)
UC	anti PD-1 plus CTLA-4	H&E, IHC	NR	NR	(92)

NR, not reported; GI, gastrointestinal cancer; CCA, cholangiocarcinoma; HNSCC, head and neck squamous cell carcinoma; STS, soft-tissue sarcomas; UC, urothelial carcinoma; IHC, immunohistochemistry; H&E: hematoxylin and eosin staining.

### 4.1 Melanoma

Immunotherapy has become an important part of the treatment for patients with advanced melanoma, but its clinical efficacy varies among patients. The majority of patients treated with ICI did not show significant clinical benefits (93). Griss et al. demonstrated that the frequency of plasmablast-like B cells in pre-therapy melanomas predicted response and survival to immune checkpoint blockade. Anti-CD20 treatment caused a downregulation of tumor-induced plasmablast-like B cells along with a significant reduction in TLSs in metastatic melanoma (94). Ding et al. confirmed that melanoma patient who responded to ICI had increased number of GC B cells and its associated Tfh cells, indicative of TLS formation, and had significantly longer OS than those with none (95). Another study by Alexandra et al. gathered a collection of tumor biopsies from melanoma patients who were receiving CTLA-4 blockade. Trichotomizing gene-expression data on the basis of the TLS signature revealed that TLS-high tumors were associated with significantly increased survival after CTLA-4 blockade (35).

### 4.2 NSCLC

With the discovery of immunotherapy, the therapeutic paradigm for patients with advanced lung cancer has fundamentally transformed (96). The revolution in immunotherapy, especially the development of ICIs, has dramatically altered the NSCLC treatment landscape (97). Brunet et al. confirmed that the presence of TLSs was not only a prognostic marker in advanced stages of NSCLC, but also a specific biomarker predictive of response to ICIs (98). Likewise, Patil et al. demonstrated that plasma-cell-rich tumors may portend OS benefit in NSCLC patients treated with ICI. The plasma cell signature was enriched in tumors with TLSs and/or lymphoid aggregates. Patients with TLS positive tumors exhibited improved OS with PD-L1 blockade (atezolizumab) (9). Wu et al. found that overexpression of most genes in TLS signature indicated a good prognosis in patients with NSCLC receiving ICI therapy (41).

### 4.3 Gastrointestinal (GI) cancers

GI cancers include esophageal, gastric, liver, biliary system, pancreatic, and colorectal cancer. A meta-analysis including 32 studies demonstrated that TLSs were significant predictor of the prognosis of GI cancer and have the potential to become biomarker for immunotherapy responses in GI cancer patients (99). Jiang et al. found that higher TLS score was correlated with a superior response to PD1 blockade therapy in patients with gastric cancer, indicating that the TLS score might be a new predictor for PD1 inhibitor therapy response (29). Shang et al. analyzed a cohort of 100 CCA patients who received first-line chemotherapy combined with ICIs to prevent postoperative recurrence. Further data indicated that a high density of intratumoral TLSs in pre-treatment tumor tissues predicted a better prognosis in patients with immunotherapy and the presence of intratumoral TLSs were associated with a prolonged OS and PFS. The study established a four-gene TLS signature as practicable biomarker for TLS identification and demonstrated that the spatial distribution and abundance of TLSs profoundly affected the prognosis and the immunotherapy response in CCA (88). Kinker et al. showed that the expression of a gene signature reflecting mature TLSs were enriched in pretreatment biopsies from PDAC patients with longer survival after receiving different chemoimmunotherapy regimens (26). Hayashi et al. derived a 4-gene TLS signature comprised of POU2AF1, LAMP3, CD79A and MS4A1 and found a significantly higher expression of this 4-gene TLS signature in responders to anti-PD-1 therapy as compared to non-responders in ESCC (89). Küçükköse et al. established a series of patient-derived organoids (PDOs) from MSI-H mCRC tumors to generate spontaneous metastasis models in mice with or without a human immune system (HIS). HIS mice with PDO-initiated MSI-H mCRC were then used to model ICI therapy. Anti-PD-1 and anti-CTLA-4 strongly reduced the growth of primary tumors and liver metastases, but peritoneal metastases were refractory to ICI. B cell influx and TLS formation were observed in ICI-responding primary tumors and liver metastases, while ICI-refractory peritoneal metastases were devoid of B cells and TLSs (100).

### 4.4 Other cancers

Some studies reported an upregulation of genes encoding chemokines involved in TLS formation in the responder patients of HNSCC, bladder cancer or soft-tissue sarcomas (STS) treated with ICIs. These high TLS signatures were associated with better OS (90, 91, 101). Meylan et al. evaluated the B cell responses within TLSs in RCC, and found IgG- and IgA-producing plasma cells infiltated into the TLS-positive tumors. RCC patients with high IgG-stained tumor cells had longer PFS and higher immunotherapy response rates (102).

## 5 The role of TLSs in evaluating neoadjuvant immunotherapy efficacy

Neoadjuvant immunotherapy is thought to produce long-term remissions through induction of immune responses and has entered standard of care in NSCLC and melanoma (103–105). Many studies have used TLSs as marker to evaluate neoadjuvant immunotherapy efficacy. Wang et al. reported that TLS‐positive TNBC patients achieved a considerable response after neoadjuvant immunotherapy with six cycles of camrelizumab. The neoadjuvant immunotherapy effect was not evident in TLS-negative patient (106). Another study by Sun et al. confirmed that TLS abundance and maturity were higher in the neoadjuvant chemoimmunotherapy group than in neoadjuvant chemotherapy group and treatment naive group in NSCLC. Patients with major pathological response (MPR) had more mature TLSs than those with non-MPR in both neoadjuvant chemoimmunotherapy and neoadjuvant chemotherapy group (87). Helmink et al. assessed the density and distribution of B cells as well as their relationship to TLSs in melanoma and RCC patients treated with neoadjuvant ICI. The density of CD20^+^ B cells and TLSs were higher in responders than in non-responders in neoadjuvant melanoma cohort, particularly in early on-treatment samples (107). Gao et al. reported the first pilot combination neoadjuvant trial with anti-PD-L1 (durvalumab) plus anti-CTLA-4 (tremelimumab) in cisplatin-ineligible urothelial carcinoma patients. They observed a higher density of TLSs in pre-treatment tumor tissues of responder patients as compared to non-responder patient. Higher density of TLSs in pre-treatment tumor tissues was correlated with longer OS (92).

## 6 Discussion

TLSs can serve as attractive biomarker for the prediction of immunotherapy response against cancer. However, heterogeneity of TLS components induced by tissue-specific factors may lead to differences in immune responses. In addition, the accumulation of regulatory immune cells within TLSs may dampen immune responses, leading to the inactivation of TLSs. Therefore, a comprehensive evaluation of TLS features would provide more accurate prediction efficacy.

Many studies have described the role of TLSs in cancer immunotherapy, but the mechanisms underlying the formation of TLSs in immunotherapy remain unclear. Rodriguez et al. showed that immunotherapy can increase TLS number and size in mouse models (63). Some studies explored the combination of cancer immunotherapy with vaccines or biomaterials to induce TLSs, probably through a mechanism involving a network of cells and chemokines (75, 108). Recent advances demonstrated that artificial or inducible TLSs (iTLSs) hold great promise to improve clinical outcomes post-immunotherapy. In addition to STING angonist, vaccine and biomaterials, iTLSs can also be achieved using stromal vascular fraction (109) or cell-free constructs (110). Besides, oncolytic virotherapies have the potential to switch cold tumors to hot tumors, and therefore could be good drivers of TLS neogenesis (111). It seems highly desirable to induce and/or augment TLS development as new aspect of cancer immunotherapy. However, the heterogeneity of TLSs in different tumors and individuals, as well as the difficulty in selection of materials for inducing TLSs still make it a challenge for iTLSs to be truly applied in clinical practice.

Unraveling the interplay between antitumor response and autoimmunity mediated by T cells, B cells and autoantibodies during TLS induction is imperative. Antitumoral immunity and autoimmune response are associated in cancer patients. A study has reported that TLSs were enriched in OMAS (an autoimmune disease) associated neuroblastomas (112). This association may be due to that an efficient TLS-induced antitumor response within the TME leads to tumor cell death and subsequent release of massive antigens that can activate autoreactive T and B cells.

In conclusion, this review revealed that the presence of TLSs indicated active antitumor immune responses and beneficial outcomes for cancer patients. The induction therapy of TLSs may provide new opportunities to improve the current immunotherapeutic treatments.

## Author contributions

CC: Writing – review & editing. XC: Writing – review & editing. MW: Writing – review & editing. FY: Writing – original draft. JY: Writing – original draft.
